# Adherence assessment of patients with metastatic solid tumors who are treated in an oncology group practice

**DOI:** 10.1186/s40064-016-1851-z

**Published:** 2016-03-03

**Authors:** Stefan Feiten, Rudolf Weide, Vera Friesenhahn, Jochen Heymanns, Kristina Kleboth, Hubert Köppler, Christoph van Roye, Jörg Thomalla

**Affiliations:** Institut für Versorgungsforschung in der Onkologie, Neversstr. 5, 56068 Koblenz, Germany; Praxisklinik für Hämatologie und Onkologie Koblenz, Neversstr. 5, 56068 Koblenz, Germany

**Keywords:** Adherence, Compliance, Metastatic solid tumor, Survey, Prescription data

## Abstract

Due to the increase of oral agents nonadherence is an emerging challenge in cancer care. We evaluated how well different assessments match and how adherence could be measured in routine care. For this purpose patients suffering from metastatic solid tumors who were treated with oral anticancer drugs in an oncology group practice were surveyed. Attending oncologists answered a questionnaire, too, and a retrospective analysis of prescription data was conducted. Caregivers who were eligible for an interview were surveyed additionally. 128 patients (70 % female) with a median age of 69 years (36–88) took part, 95 % of all approached patients. 56 % suffered from metastatic breast cancer, 44 % from other metastatic solid tumors. 65 caregivers (60 % female) with a median age of 62 years (21–82) were interviewed as well. Patients were assessed in 84 % as very reliable in medication-taking by their oncologists. This high adherence rate was supported by patients, caregivers and prescription data. However, concordance between assessments of patients, caregivers and oncologists was not substantial. Our method of considering different perspectives to assess adherence has to be improved and validated but could help to evaluate adherence with oral cancer therapy in routine care.

## Background

Drugs do not work in patients who do not take them (Foulon et al. 2011). Adherence with oral drugs, defined as the extent to which patients take medications as prescribed (Barron et al. 2007; Osterberg and Blaschke 2005), is a challenge of increasing interest (Ziller et al. 2009). Consequences of nonadherence are far-reaching, both from an economic standpoint and in terms of morbidity and mortality (Dezii 2009; Puts et al. 2014). Adherence rates of less than 80 % were associated with poorer survival in breast cancer patients taking tamoxifen (McCowan et al. 2008), similar results were found in various studies on chronic myeloid leukemia (Ibrahim et al. 2011; Marin et al. 2010; Al-Barrak and Cheung 2013). The monetary waste associated with nonadherence is estimated for the US between $100 billion (Dezii 2009) and $300 billion a year (Foulon et al. 2011; DiMatteo 2004). Patients are optimally adherent if no doses are missed, no extra doses are taken, and no doses are taken in the wrong quantity or at the wrong time (Ruddy et al. 2009). The terms adherence and compliance can be used synonymously (Ruddy et al. 2009); persistence, defined as continuing treatment for the prescribed duration (Hohneker et al. 2011), should be distinguished (Ruddy et al. 2009; Cramer et al. 2008).

Despite a substantial number of studies in the past decades (DiMatteo 2004; Vermeire et al. 2001) there is still no “gold-standard” method (Foulon et al. 2011; Grymonpre et al. 2006; Vermeire et al. 2001; Banning 2008, 2012; Waterhouse et al. 1993; Escalada and Griffiths 2006) in measuring adherence and only a combination of measures can maximize accuracy (Osterberg and Blaschke 2005; Wang et al. 2004; Zahrina et al. 2014).

Self-report instruments are cheap, easy to use and uncomplicated to score (Foulon et al. 2011) but fraught with difficulties, including recall issues and reluctance to admit nonadherence (Palmieri and Barton 2007; Ruddy et al. 2009; Wang et al. 2004; Figueiredo Junior and Forones 2014). For pill counts patients are required to return unused pills at each next visit (Foulon et al. 2011), but like self-reports they may be inaccurate and open to manipulation (Palmieri and Barton 2007; Ruddy et al. 2009). Rates of prescription refills are objective and easy to obtain but a prescription refill is not equivalent to ingestion of medication (Osterberg and Blaschke 2005). Drug levels in blood or urine or other physiological parameters may be more effective, but are subject to individual differences because of pharmacokinetic factors (Palmieri and Barton 2007) and for most oral anticancer drugs the available markers are not fully validated or sensitive and reliable enough to assess adherence (Foulon et al. 2011). Electronic monitoring may provide the best estimate of patient adherence (Foulon et al. 2011), however it is expensive and not always feasible in daily practice (Ruddy et al. 2009; Foulon et al. 2011) and an open container does not equal tablet ingestion (Palmieri and Barton 2007). In sum, there are serious problems with each method for generating valid and reliable data to give an accurate estimate of extent of adherence (Vermeire et al. 2001).

Due to differences in measurement and research context wide variations in adherence estimates and outcomes can be found in the literature (DiMatteo 2004). According to World Health Organization (WHO) adherence to long-term treatments for chronic conditions is estimated to be only 50 % (World Health Organization http://www.who.int/chp/knowledge/publications/adherence_report/en/#). DiMatteo (2004) conducted a meta-analysis with 569 studies covering a research period of 50 years and found a median adherence of 76 % and a range from 5 to 100 %; studies involving cancer patients had a mean adherence of 79 %. Adherence rates of patients using oral anticancer agents ranging from 20 to 100 % have been reported elsewhere (Ruddy et al. 2009; Partridge et al. 2002, 2003).

In the longer term, oral cancer therapy may well be the rule rather than the exception (O’Neill and Twelves 2002). Given this shift and the often required longer term use (Weingart et al. 2008; Barton 2011), adherence is an emerging challenge in cancer care (Barton 2011; Owusu et al. 2008). In oncology, adherence has been investigated most often in breast cancer patients (Barron et al. 2007; Ziller et al. 2009; Banning 2012; Waterhouse et al. 1993; Owusu et al. 2008) or in patients suffering from chronic myeloid leukemia (Ibrahim et al. 2011; Marin et al. 2010; Al-Barrak and Cheung 2013; Eliasson et al. 2011). Breast cancer patients have been assessed typically in an early stage of disease and there is only little information on the adherence with palliative treatments (World Health Organization http://www.who.int/chp/knowledge/publications/adherence_report/en/#). Nevertheless some studies have been recently published investigating adherence with oral chemotherapy or targeted agents in patients suffering from metastatic breast cancer (Figueiredo Junior and Forones 2014; daCosta DiBonaventura et al. 2014), metastatic colorectal cancer (Figueiredo Junior and Forones 2014), metastatic castration-resistant prostate cancer (Smith et al. 2015), metastatic renal cell cancer (Wolter et al. 2012), advanced non-small cell lung cancer (Timmers et al. 2015) and advanced gastric cancer (Yagasaki et al. 2015).

The objectives of the present study were as follows:

1. Assessment of medication-taking
2. Assessment of adherence with oral anticancer drugs
3. Concordance of adherence assessments of patients, caregivers and oncologists

To the best of our knowledge this is the first evaluation of adherence in cancer patients with metastatic solid tumors who are treated in an oncology group practice.

## Methods

All patients who suffered from a metastatic solid tumor and received oral anticancer therapy (cytotoxic or hormonal) were identified. Eligible patients were informed by their oncologist and gave written informed consent. The interviews were carried out by study nurses and lasted 10–15 min. The standardized computer-assisted questionnaire covered the following areas: handling of and attitudes to drugs in general, tolerability and efficacy of cancer treatment, importance of therapy for the own health, self-reported adherence and handling of adherence problems.

For all eligible caregivers additional interviews with the main topic patient’s reliability with medication-taking were conducted. Caregivers had been identified by the patients during their interviews with the help of an open-ended question as to persons who support them in medication-taking.

A written survey of the attending oncologists with the topics tolerability and efficacy of the prescribed anticancer drug and the reliability of the patient with medication-taking was performed for all investigated patients.

Tolerability and efficacy of anticancer drugs were evaluated by patients and attending oncologists with the help of a Likert-scale ranging from 1 to 5. “1” meant that the therapy was “very well tolerated” or “very effective” and “5” that the treatment was “not at all” tolerable or effective. Reliability of patients in medication-taking was assessed by the attending oncologist and the caregiver using a five-point scale ranging from “1” “very reliable” to “5” “not reliable at all”.

In addition, a retrospective analysis of the medical treatment data was carried out for all patients using medical records and checking prescriptions for deviation from the documented treatment regimen. Anticancer drugs were thought to be prescribed only by oncologists. As an assessment period the previous 6 months were used.

The above mentioned interviews of patients, caregivers and attending doctors were developed according to existing literature and used in a small number of patients in terms of a pre-test; they are not validated so far.

The issue of adherence was thus classified from 3 or 4 different and independent perspectives per patient:

1. survey of patients
2. survey of attending doctors
3. survey of caregivers (if possible)
4. retrospective analysis of prescription data

Statistical analyses were performed using SPSS 19. Frequencies, medians and means were calculated to describe the data. Concordance analyses of caregivers’ and oncologists’ assessments were conducted using Lin’s Concordance Correlation Coefficient and Cohen’s Kappa. These analyses had to be limited to patients whose caregivers had carried out an interview. Associations between patients’ self-reports and assessments of caregivers and attending doctors were evaluated with Chi square trend tests.

Missing values occurred only sporadically; if so, fewer cases were included in the analyses. The entire project had an exploratory character, specific hypotheses were not verified.

## Results

A total of 135 patients were approached, 95 % participated. Overall 128 patients (70 % female) with a median age of 69 years (36–88 years) were interviewed. 56 % suffered from metastatic breast cancer, 44 % from other metastatic solid tumors.

65 caregivers (60 % female) with a median age of 62 years (21–82 years) could be questioned as well. Caregivers were partners in 69 % and children in 23 % and lived in 69 % of cases in the same household as the patient. Patients’ and caregivers’ characteristics are depicted in Table 1.

**Table 1:** Patients’ and caregivers’ characteristics

Patients (N = 128)	
Sex	
Female	n = 89 (70 %)
Male	n = 39 (30 %)
Age at the time of the interview	
Median (range)	69 years (36–88)
Diagnoses	
Metastatic breast cancer	n = 72 (56 %)
Metastatic prostate cancer	n = 18 (14 %)
Metastatic renal cell cancer	n = 9 (7 %)
Other metastatic solid tumors	n = 29 (23 %)
Administered oral anticancer drugs	
Hormonal therapies	
Letrozole	n = 17 (13 %)
Abiraterone acetate	n = 11 (9 %)
Exemestan	n = 11 (9 %)
Anastrozole	n = 10 (8 %)
Exemestane/everolimus	n = 7 (5 %)
Other hormonal therapies	n = 13 (10 %)
Oral chemotherapies	
Capecitabine	n = 27 (21 %)
Other chemotherapies	n = 7 (5 %)
Capecitabine + hormonal therapy	n = 2 (2 %)
Signal transduction inhibitors	
Pazopanib	n = 7 (5 %)
Other signal transduction inhibitors	n = 15 (12 %)
Temozolomide + vemurafenib	n = 1 (1 %)
Level of education	
No educational qualification	n = 2 (2 %)
Secondary school	n = 106 (83 %)
A levels	n = 12 (9 %)
University	n = 8 (6 %)
Employment status	
Employed	n = 16 (13 %)
Retired	n = 99 (77 %)
Not employed	n = 13 (10 %)
Caregivers (N = 65)	
Sex	
Female	n = 39 (60 %)
Male	n = 26 (40 %)
Age at the time of the interview	
Median (range)	62 years (21–82)
Relationship to the patient	
Partner	n = 45 (69 %)
Child	n = 15 (23 %)
Other	n = 5 (8 %)
Living in the same household	
Yes	n = 45 (69 %)
No	n = 20 (31 %)

### Assessment of medication-taking

52 % of patients took their medications straight from the packaging. 43 % used a pill dispenser and prepared pills most often for a week (22 %) or a day (20 %) in advance. 31 % were supported by another person, usually by reminding (18 %) and/or by preparation in a pill dispenser (16 %). Assessments of trained interviewers showed that 75 % of patients were able to name their cancer medications correctly and completely without any help. After presentation of personal prescription sheets additional 17 % identified their anticancer drugs properly and completely, in 7 % the responses remained incomplete and/or incorrect. According to treatment files, patients took 7 (1–19) different medications in median, the median number of oral anticancer drugs was 1 (1–4).

Tolerability of anticancer drugs was rated quite similarly by patients (1.8) and physicians (1.7). Men (1.7) and patients receiving hormonal therapy (1.6) indicated a slightly better tolerance than women (1.9) and patients receiving oral chemotherapy (1.9). 35 patients could not answer the question of therapeutic effectiveness. The remaining 93 patients assessed treatment efficacy as high (1.6). Men (1.3) and patients receiving hormonal therapy (1.4) perceived a better therapeutic efficacy than the corresponding control groups (1.7 and 1.9).

### Assessment of adherence with oral anticancer drugs

Deviation between number of prescribed pills and the, according to the treatment regimen, necessary number was less than 10 % in 85 % of patients, between 10 and 20 % in 8 % and more than 20 % in 6 % of patients. In 19 patients prescription rates could not be calculated exactly due to lacking data. 88 % of respondents indicated to take the prescribed medication always or almost always exactly as prescribed.

Patient’s reliability in medication-taking was assessed both from the attending oncologist and from the caregiver using a five-point scale. Oncologists estimated the patients in 84 % of cases as very reliable, with a mean of 1.3 for all patients. In further analyses no differences between age groups, cancer diagnoses, type of prescribed medication and sex could be found. Patients living alone (1.6) were assessed less adherent than patients who lived together with another person (1.2). Mean values of subgroups can be depicted in Table 2.

**Table 2:** Reliability in medication-taking—assessed from the attending oncologist (mean values)

Total (N = 128)	1.3
5 point scale ranging from “1” “very reliable” to “5” “not reliable at all”	
Patients’ characteristics	
Sex	
Female (n = 89)	1.3
Male (n = 39)	1.2
Age groups	
65 years and younger (n = 54)	1.3
66 years and older (n = 74)	1.2
Diagnoses	
Metastatic breast cancer (n = 72)	1.2
Other metastatic solid tumors (n = 56)	1.3
Type of prescribed medication	
Hormonal therapy (n = 69)	1.3
Oral chemotherapy (n = 34)	1.3
Signal transduction inhibitors (n = 22)	1.2
Living in the household	
Living alone (n = 23)	1.6
Living together with another person (n = 97)	1.2

Assessments of adherence from different perspectives and analyses of treatment files are summarized in Table 3.

**Table 3:** Adherence in medication-taking—assessed from different perspectives

Assessment of patient	
Taking medication always or almost always as prescribed	n = 113 (88 %)
Deviation in prescription data (n = 109)	
Less than 10 %	n = 93 (85 %)
10–20 %	n = 9 (8 %)
More than 20 %	n = 7 (6 %)
5 point scale ranging from “1” “very reliable” to “5” “not reliable at all”:	
Assessment of oncologists (n = 63)	
Mean value	1.1
Assessment of caregivers (n = 63)	
Mean value	1.2

### Concordance of adherence assessments of patients, caregivers and oncologists

Lin’s concordance correlation coefficient failed to show a concordance in assessments of attending doctors and caregivers; the computed coefficient of .3248 has to be regarded as poor. Inter rater agreement according to Cohen’s Kappa was .144 which cannot be regarded as substantial, too. Sensitivity was 86 %, specificity 50 %.

Chi square trend tests showed no significant associations between patients’ self-reports and assessments of attending doctors (p = .081) and caregivers (p > .999).

## Discussion

### Medication-taking behavior and adherence in general

Nonadherence to oral medication is associated with numerous behavioral, psychosocial and other risk factors (Zeber et al. 2013), it may be intentional or non-intentional (Vermeire et al. 2001; Banning 2008; Eliasson et al. 2011). In our population patients with a median age of 69 years had to take seven different oral drugs in median and only 31 % of these patients received support by a caregiver. One can easily imagine that single doses could be missed or that overdosing could be an issue. But a current review concluded that demographic variables, especially age (Ruddy et al. 2009) and disease factors are poor indicators of adherence (Vermeire et al. 2001). No relationship between adherence and the use of a reminder method could be found in a prospective study (Timmers et al. 2015). Nonetheless, our results seem to support the finding that patients living alone are less adherent (Timmers et al. 2014).

### Estimation of adherence with oral anticancer drugs

Cancer patients are often thought to be highly motivated by the gravity of their disease, with “too much to lose” by being nonadherent (Ziller et al. 2009; Waterhouse et al. 1993; Zahrina et al. 2014; Winterhalder et al. 2011). Recent studies reported adherence rates between 82.6 and 96.8 % in patients suffering from metastatic disease (Figueiredo Junior and Forones 2014; Smith et al. 2015; Timmers et al. 2015).

Our self-report data show an adherence-level of 88 % of patients indicating to take their prescribed anticancer medication always or almost always exactly as prescribed. Oncologists assessed the patients in 84 % as very reliable, with a mean of 1.3 on the five-point scale. The deviation between number of prescribed and necessary number of pills was more than 20 % in only 6 % of patients. In sum, our results support the conclusion that adherence with oral anticancer therapy seems to be higher than for medical treatment in general (Zahrina et al. 2014). There are reasons to believe that this comparable high level of adherence contributes to favorable outcomes, e.g. in patients with metastatic breast cancer (Weide et al. 2014).

However, most studies have shown a number of patients who may need specific interventions to ensure adherence, too (Figueiredo Junior and Forones 2014). Based on different and independent data sources in our population a small group of about 5–10 % of patients can be found who are at risk for nonadherence. For instance 6 % of patients had a discrepancy between number of prescribed and number of necessary pills of more than 20 %.

### Measuring adherence with the help of different perspectives: lacking concordance

Several methods can be used to detect nonadherence and for each advantages and limitations can be defined (Foulon et al. 2011). The simplest method for physicians to monitor adherence may be self-report (Ruddy et al. 2009) and self-reports are often the only means available in routine care even though their accuracy and agreement with other data sources remains questionable (Wang et al. 2004). But an additional perspective of caregivers seems to be little helpful, especially with regard to identification of patients who are less reliable, since we found a specificity of only 50 %. Furthermore we experienced difficulties in using routine care data to assess adherence in medication-taking reliably. Prescription data remained unclear for various reasons. Major problems were additional prescriptions, i.e. aromatase inhibitors or tamoxifen by gynecologists, and undocumented changes in regimen.

Nevertheless it is necessary for clinicians to detect patients with low adherence as early as possible (Simons et al. 2011). We therefore suggest to screen for nonadherent patients with the help of self-report questionnaires and to evaluate nonadherence in more detail for patients who may be at risk. In this sense our approach could be feasible, questionnaires were short and easy to administer and notwithstanding a lacking “gold-standard” measure we believe that it is possible to assess adherence conveniently. Furthermore, given the Hawthorne effect, a systematic monitoring of patient pill-taking may be as well an effective way to improve adherence and persistence (Ruddy et al. 2009).

### Methodological considerations

Overall there is only little information on the adherence with palliative treatments of cancer patients (World Health Organization 2003http://www.who.int/chp/knowledge/publications/adherence_report/en/#), since they may have a different motivation to take their medication than those receiving an adjuvant therapy (McCowan et al. 2008). The strength of our project is the population of unselected real-life patients who suffer from metastatic solid tumors. To our knowledge this is the first assessment of adherence in patients who are treated in an oncology group practice. According to current data approximately 600,000 cancer patients are treated annually in specialists’ practices in Germany (Berufsverband der Niedergelassenen Hämatologen und Onkologen in Deutschland e.V 2012http://www.bnho.de/startseite.html).

We believe that in this setting it is feasible to assess adherence, only a small expenditure of additional time is necessary. But there are serious limitations which have to be considered. First of all, a retrospective analysis of prescription data is difficult and there are too many cases in which adherence could not be assessed adequately due to missing data. Secondly, we could only manage to survey about 50 % of all caregivers; analyses of concordance thus were limited to only a half of all investigated patients. Further limitations are the sample size of 128 patients and the cross-sectional unicenter design which made it impossible to address the question of persistence.

## Conclusions

Patients suffering from metastatic solid tumors who are treated in an oncology group practice seem to be highly adherent with oral antineoplastic therapies. Nevertheless, a small group of about 5–10 % of patients exists who are not fully adherent.

There is no substantial concordance in adherence assessments of patients, attending physicians and caregivers. Retrospective analyses of prescription data are difficult due to lacking data. Still our method of considering different perspectives to assess adherence seems to be suitable in routine care but it has to be improved and validated in larger and more reliable trials.
